# TRNT1 missense mutations define an autoinflammatory disease characterized by recurrent fever, severe anemia, and b-cell immunodeficiency

**DOI:** 10.1186/1546-0096-12-S1-O21

**Published:** 2014-09-17

**Authors:** Ivona Aksentijevich, Qing Zhou, Angeliki Giannelou, Anna Sediva, Deborah Stone, Sergio Rosenzweig, Jehad Edwan, Martin Pelletier, Stoffels Monique, Lucie Šrámková, Amanda Ombrello, Karyl Barron, Daniel Kastner

**Affiliations:** 1 National Human Genome Research Institute, Bethesda, USA; 2 National Institute for Arthritis and Musculoskeletal and Skin Diseases, Bethesda, USA; 3 University Hospital Motol, Prague, Czech Republic; 4 National Institutes of Health, Bethesda, USA; 5 National Institute of Allergy and Infectious Diseases, Bethesda, USA

## Introduction

We observed a syndrome characterized by recurrent fever, severe anemia, gastrointestinal symptoms, and a spectrum of immunologic and neurologic symptoms in five children from four unrelated families. Neurologic manifestations ranged from mild developmental delay to nystagmus, spasticity, optic nerve atrophy, and sensorineural hearing loss. Sideroblastic anemia was identified by bone marrow biopsies in two of the children.

## Objectives

We suspected a genetic cause because of early onset symptoms.

## Methods

We performed whole-exome sequencing in three unrelated families and candidate gene sequencing in one patient with a similar phenotype. Cytokine profiling, flow cytometry, mitochondria-function and ribosomal assembly related experiments were performed on samples from patients. We used morpholino-mediated knockdowns in zebrafish to study protein function.

## Results

After filtering for novel and rare variants (allele frequency <1:1000) and homozygous recessive inheritance in one consanguineous family, we observed that three patients carried missense mutations in *TRNT1*, encoding tRNA nucleotidyl transferase, CCA-adding, 1. By additional exome and Sanger sequencing we found two other patients with mutations in *TRNT1*. All disease-associated mutations affect highly conserved amino acid residues and are predicted to be damaging to the protein function. The first family from Saudi Arabia had two affected daughters, both homozygous for the p.H215R mutation; the second family of mixed Czech and British background had one affected son, carrying a compound heterozygous p.I223T/p.D163V mutation; two families of mixed European ancestry from the US each had one affected daughter, both compound heterozygous for a p.R99W/p.D163V mutation. Two out of five patients died. The p.H215R mutation was not found in any public database or in 1061 Arabian control DNA samples. The three Caucasian mutations are either novel, or found at a very low allele frequency, consistent with recessive inheritance. Cytokine profiling revealed increased IL-6 serum levels in 2 patients suggesting that their inflammation may be driven by the IL-6 cytokine. Preliminary analysis of two patients who presented with severe B-cell immunodeficiency suggests that the paucity of B-cells is caused by the abnormal proliferation and maturation of B-cells. Knockdown of the zebrafish *TRNT1* homologue caused hydrocephaly, defects in tail development, anemia and a reduction in the number of hair cells present in the lateral line, which subserves functions of the inner ear in zebrafish.

## Conclusion

Missense mutations in *TRNT1* are associated with an autoinflammatory disease manifesting with fevers, transfusion dependent anemia, gastrointestinal symptoms, immunologic, and neurologic symptoms. The TRNT1 enzyme catalyzes the addition of the CCA terminus to the 3-prime end of tRNA precursors and is essential for protein biosynthesis. This phenotype is distinct from other autoinflammatory disorders for the reason that the mutated protein have a profound effect on multiple cells and organs. This likely explains a broad spectrum of features in these patients that are consitent with mitochondrial phenotypes.

## Disclosure of interest

None declared.

